# Abnormal emotion processing, but intact fairness and intentionality considerations during social decision-making in schizophrenia

**DOI:** 10.3389/fpsyg.2015.01058

**Published:** 2015-07-23

**Authors:** Javier de la Asuncion, Lise Docx, Bernard Sabbe, Manuel Morrens, Ellen R. A. de Bruijn

**Affiliations:** ^1^Collaborative Antwerp Psychiatric Research Institute, Faculty of Medicine and Health Sciences, University of AntwerpAntwerp, Belgium; ^2^Psychiatric Center Brothers AlexiansBoechout, Belgium; ^3^University Psychiatric Center St. Norbertushuis, DuffelBelgium; ^4^Department of Clinical Psychology, Leiden Institute for Brain and Cognition, Leiden UniversityLeiden, Netherlands

**Keywords:** schizophrenia, social decision-making, fairness, intentionality, emotions, emotion processing, Ultimatum Game

## Abstract

Schizophrenia is a severe mental disorder that is highly characterized by social cognitive impairments. Most studies investigating these impairments focus on one specific social domain such as emotion recognition. However, in daily life, processing complex social situations relies on the combination of several social cognitive and affective processes simultaneously rather than one process alone. A modified version of the economically based Ultimatum Game was used to measure the interplay between fairness, intentionality, and emotion considerations during social decision-making. In this task, participants accept or reject fair and unfair monetary offers proposed intentionally or unintentionally by either angry, happy, neutral, or sad proposers. Behavioral data was collected from a group of schizophrenia patients (*N* = 35) and a group of healthy individuals (*N* = 30). Like healthy participants, schizophrenia patients differentiated between fair and unfair offers by rejecting unfair offers more compared to fair offers. However, overall patients did reject more fair offers, indicating that their construct of fairness operates within different margins. In both groups, intentional unfair offers were rejected more compared to unintentional ones, indicating a normal integration of intentionality considerations in schizophrenia. Importantly, healthy subjects also differentiated between proposers’ emotion when rejecting unfair offers (more rejections from proposers depicting angry faces compared to proposers depicting, happy, neutral, or sad faces). Schizophrenia patients’ decision behavior on the other hand, was not affected by the proposers’ emotions. The current study thus shows that schizophrenia patients have specific problems with processing and integrating emotional information. Importantly, the finding that patients display normal fairness and intentionality considerations emphasizes preservation of central social cognitive processes in schizophrenia.

## Introduction

The last two decennia research in schizophrenia has shifted its focus intensively from positive (e.g., hallucinations, delusions) and negative symptoms (e.g., flattened affect, anhedonia; see Andreasen et al., 1994) to social cognitive deficits. An important reason for this shift of interest relates to findings indicating that these social cognitive deficits are highly predictive for patients’ functional outcomes (Brekke et al., 2005). However, most studies investigating social cognitive processes in populations suffering from schizophrenia, rely on basic, relatively quick, and automated cognitive processes as measured in, for example, emotion-recognition tasks. Results on such tasks suggest that schizophrenia patients experience problems in the perception of emotional material, however, the specificity, extent, and nature of the deficits are unclear (Edwards et al., 2002). Yet, in order to cope with different social situations higher-order social cognitive processes such as fairness or intentionality considerations are also essential skills. These higher-order social cognitive processes are often very complex as they rely on combinations of different (social) cognitive and affective abilities (see Pinkham, 2014 for a review on the core social cognitive domains in schizophrenia). For instance, when someone wants to buy something, one addresses his or her *social knowledge* about the context and environment where he or she is in (e.g., buying a souvenir at an exotic holiday spot or buying food at your local market), but also considers the other person’s personal inferences (*Theory of Mind*; e.g., is someone selling for personal profit or for charity purposes), and uses basic *emotion-recognition* processes (e.g., is the person you are buying from happy or angry). Together with someone’s attitude and personality traits, these social cognitive processes contribute to one’s judgments and decisions in many daily life situations, and importantly, also contribute to others’ perception of yourself.

During such complex social interactions, requiring reciprocity an trust (Wischniewski and Brüne, 2011), and emotion regulation processes (van der Meer et al., 2009), schizophrenia patients tend to respond differently in comparison to healthy persons. For instance, while testing the appreciation of moral standards, older studies showed that schizophrenia patients less often choose humanitarian responses to moral problems, and instead more often choose authoritarian and self-protective options (Johnson, 1960), or even adjust their moral decisions according to the concepts of power, status, and possessions, rather than equality and reciprocity (Benson, 1980). Unfortunately, this line of research might have contributed to the stigmatization of schizophrenia patients as being immoral beings in a way that during the recent past studies about social norms and moral values in schizophrenia have been disregarded (Wischniewski and Brüne, 2011). Yet, to our knowledge, only one study so far by Wischniewski and Brüne (2011) addressed the possibility that deviating social norms and standards in schizophrenia might also relate to these patients’ higher prevalence of being victimized (Hodgins et al., 2009), treated unfairly, or being bullied (Trotta et al., 2013). All factors known for their negative interferences with these patients’ functional outcomes (Hodgins et al., 2009).

Recently, higher-order moral judgments in humans have often been investigated by using economic games like the Ultimatum Game (UG) showing that moral judgment is universal and deeply rooted in human nature. During the UG, two players split a certain amount of money. One player plays the role of the proposer, the other player acts as the responder. The proposer decides how the money is split while the responder either accepts or rejects the proposed offer. When the responder accepts the offer, the amount of money is divided accordingly. However, if the responder disagrees with the proposed split and rejects the offer, neither player receives anything. Although the most profitable strategy from an economical perspective would be to accept even the smallest offers, studies show that healthy individuals tend to use different strategies based on fairness and other emotional aspects, rather than rational inferences. Therefore, unfair offers (30% or less from the total amount) are more likely rejected in comparison to fair splits (50%; Nowak et al., 2000).

In schizophrenia, the impairments of certain higher-order social cognitive abilities such as social norms and values (Wischniewski and Brüne, 2011) might be related to these patients’ known cognitive (e.g., executive functioning; Nuechterlein et al., 2012) and social cognitive (e.g., emotion processing; Green et al., 2012) dysfunctions. Yet, across several UG studies schizophrenia patients depicted an inconsistent decision pattern. For instance, Agay et al. (2008) was the first to report that schizophrenia patients, when acting as responders, showed no difference in rejection rates compared to healthy controls. However, Wischniewski and Brüne (2011) showed that schizophrenia patients were likely to accept more unfair offers when compared to healthy individuals. The latter finding is in line with a study reporting that unfair offers are also more likely to be accepted by individuals with high schizotypal traits (van’t Wout and Sanfey, 2011). Yet, another study reported that schizophrenia patients accepted more unfair offers and rejected more fair offers in comparison with healthy controls (Csukly et al., 2011). Above that, they also found that these patients’ acceptance/rejection behavior was not affected by the emotion of the proposer, whilst healthy controls accepted more offers proposed by happy individuals than angry individuals (depicted on a photograph).

While these studies mainly focus on fairness considerations and the reactions toward the direct outcomes of the proposed offers, they do not allow to measure intentional variations of the proposed offers. Yet, as stated before, social decision-making is a complex process requiring the integration of several cognitive and social cognitive abilities working together toward a final decision. Therefore, a modified version of the UG has previously been developed in a way that each proposed offer is contrasted against another possible alternative offer that has not been chosen (Falk et al., 2003; Güroğlu et al., 2010). Using this method, the responder can weigh the proposer’s offer against an alternative offer that is either more fair, more unfair, or the same (no alternative). Studies that used this modified version of the UG showed that healthy individuals reject unfair offers more often in the presence of an unselected fair alternative compared to situations where the proposer could only choose between two equally unfair offers (no-alternative; Falk et al., 2003; Güroğlu et al., 2009, 2010; Radke and de Bruijn, 2012; Radke et al., 2012). This emphasizes that fairness considerations are not only depending on the direct profitable outcomes, but also depend on contextual factors and the intentions they signal (Falk et al., 2008).

In schizophrenia, this method was recently also used in a crossover study directly comparing smoking and non-smoking patients after administration of a placebo, 1 or 2 mg of nicotine (Quisenaerts et al., 2013). Results showed that smoking patients’ decisions were affected by the unchosen alternative offer (context) in the expected pattern described above. Non-smoking patients’ on the other hand did not dissociate between the different alternatives. They did show, however, a normalized effect of context after administration of 1 mg of nicotine. The authors argued that this normalizing effect in non-smokers might be related to the inverted U shape nature of the cognitive enhancing properties of nicotine. However, intentionality can also be defined by the emotion one expresses. This has been demonstrated by Schreiner et al. (2010) and Csukly et al. (2011) who showed that healthy individuals accepted more offers from happy proposers than from angry proposers.

Crucially, in daily life, these various determinants of social decision-making are combined and have to be processed simultaneously, thus complicating the task tremendously. A recent study aimed at targeting this complexity by combining fairness, contextual, and affective variables into a modified UG (Radke et al., 2013). The results of this study showed that both healthy controls’ and depressed patients’ rejection rates were highest when the unfair treatment was clearly intentional, so when paired with a fair alternative and when offered with an angry expression. Overall rejection rates were, however, larger in the patient group.

Because of the contradictory findings on social decision-making in schizophrenia research so far, it is important to investigate the involved processes as they occur simultaneously and need to be integrated for adequate decision-making. Following Radke et al. (2013), we therefore used a modified version of the UG that allowed us to disentangle fairness, intentionality, and emotion considerations. Based on the findings of Quisenaerts et al. (2013), we first hypothesize that like healthy individuals, schizophrenia patients’ decisions will be affected by intentionality, i.e., the unchosen alternative offer. Second, as reported in the study of Csukly et al. (2011), we expect that schizophrenia patients’ decisions are less affected by the proposers’ emotions. Third, because of the mixed outcomes regarding acceptance and rejections rates of fair and unfair offers in previous UG studies we also hypothesize that schizophrenia patients will show aberrant behavior when considering fair versus unfair offers by either rejecting more fair offers or accepting more unfair offers than healthy controls. Given the complexity of the study design and the absence of any previous studies investigating context effects such as emotion and intentionality during fairness considerations in schizophrenia patients, we refrained from formulating specific hypotheses about modulatory influences of these contexts on group differences in rejection rates.

## Materials and Methods

### Participants

The patient group consisted of 37 schizophrenia patients (25 inpatients) recruited from three different Belgian psychiatric centers (PC Sint-Norbertus Duffel: *N* = 23; PC Sint-Amadeus Mortsel: *N* = 11; PC Brother Alexians Boechout: *N* = 3) diagnosed using the Structured Clinical Interview for DSM-IV Axis I disorders (SCID-I; First et al., 2002). Patients with current depression or a recent history of substance use disorder (6 months) were excluded. All patients were stable on antipsychotic medication for at least 2 weeks. Fifteen patients received monotherapy with an atypical antipsychotic, one received conventional neuroleptic monotherapy and 21 patients were on polytherapy (14 patients were treated with a combination of atypicals and 9 received a combination of an atypical antipsychotic and a conventional neuroleptic). In order to control for differences in medication, chlorpromazine levels are calculated (cf., Kroken et al., 2009) based on the patients’ medication profiles. Besides antipsychotic medication, some patients were treated with mood stabilizers (*N* = 8), antidepressants (*N* = 14), benzodiazepines (*N* = 7), and/or anticholinergics (*N* = 4). Severity of the positive and the negative symptoms were rated during a semi-structured interview using the Scale for the Assessment of Negative Symptoms and the Scale for the Assessment of Positive Symptoms (SANS and SAPS; Andreasen, 1983, 1984).

The control group consisted of 30 healthy individuals that were matched for age and gender with the patient group. The study was approved according to the latest Declaration of Helsinki by all the local ethical committees of the participating centers and all participants gave their written informed consent.

Since high rejection rates of fair offers clearly indicate a lack of understanding the task objective, participants who rejected 75% or more fair offers were excluded from analyses, which resulted in the exclusion of two patients. This left us with a group of 35 schizophrenia patients and 30 healthy controls (see **Table 1** for group characteristics).

**Table 1:** Clinical and sociodemographic data.

	Controls	Patients	*T*	*X*^2^	*p*
	*N* = 30	*N* = 35			
Age	29.6 (9.3)	31.2 (8.3)	0,736		0.464
Gender (m/f)	26/4	32/3		-0,613	0.540
Duration of illness (years)		7.6 (7.0)			
SAPS		14.3 (12.0)			
SANS		33.6 (16.4)			
CDS		0.7 (1.5)			
Chlorpromazine equivalent		556 (371)			

*SAPS, Scale for the Assessment of Positive Symptoms; SANS, Scale for the Assessment of Negative Symptoms; CDS, Calgary Depression Scale. Values shown are absolute or means with SD between parentheses.*

### Material and Procedure

**FIGURE 1:**
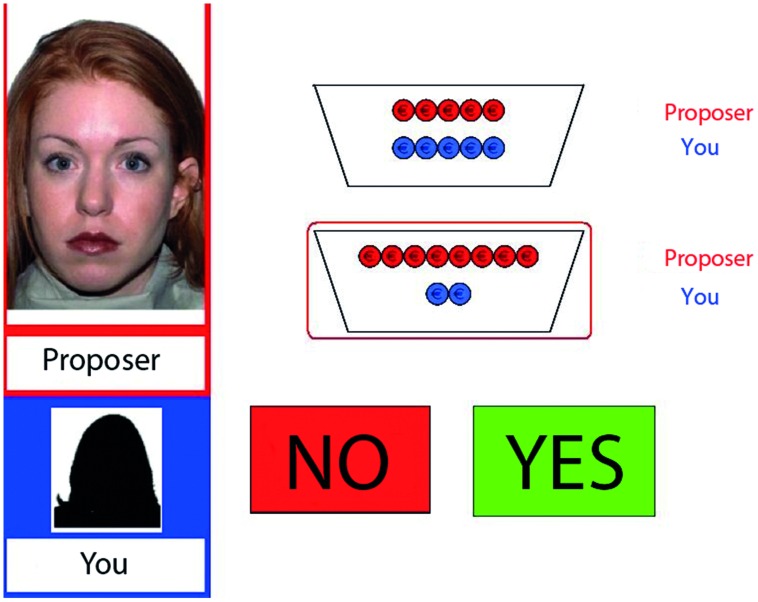
**Display of the decision phase in the fair-alternative condition with a neutral proposer.** On the left, name and picture of the proposer (here “Proposer”) and the name of the participant (here “You”) are shown. Red and blue coins specify the two potential distributions. The selected offer is encircled in red. The participant has to decide whether to accept (“YES”) or reject (“NO”) the offer.

Stimuli were presented using E-Prime 2.0 software (Psychology Software Tools Inc, 2012, Pittsburgh, PA, USA) that was programmed with a modified version of the UG (**Figure 1**) in which participants played the role of the responder (cf. Radke et al., 2013). They were told to be playing against the saved data of different proposers who previously participated in this game. On each of the 64 trials, a picture of a different proposer with his or her fictive name was shown in the upper left part of the screen. These pictures were derived from different databases (Lundqvist et al., 1998; Ebner et al., 2010).

Each trial started with a fixation cross (1000 ms), followed by a presentation of two available monetary distributions (1000 ms). Then, the proposer’s selected offer was surrounded by a red rectangle (1000 ms). Subsequently, while the selection remained visible, “yes” and “no” buttons were presented as depicted in **Figure 1**. The participants had unlimited time to respond by pressing one of the two assigned keys on a keyboard. Participants’ response remained visible for 2000 ms before the next trial started. The position of unfair offers and the proposer’s gender were counterbalanced. In contrast with the participants’ belief, all choices were computer-generated and randomly presented.

By pressing the “yes” or “no” key, participants either accepted or rejected the proposer’s offer. Acceptance resulted in an outcome according to the proposed split while rejection resulted in a complete loss for both. To assure the participants’ motivation, they were informed that every trial could influence their financial outcome at the end of the task since several trials were randomly chosen in order to compute their personal profit. Moreover, participants were also instructed about the influence of their decisions on the proposers’ profit that would be paid to them after all data of the responders had been collected. In fact, the payoff was set around 2.50 Euro, so that all participants received an equal amount.

### Design and Analyses

The task consisted of 64 trials. On 40 trials the unfair offer (8:2) was selected against (i) a hyperfair (2:8) alternative (8 trials, 2 per emotion), (ii) a fair (5:5) alternative (16 trials, 4 per emotion), or (iii) no alternative (8:2; 16 trials, 4 per emotion). On 16 trials a fair offer was selected against an unfair alternative (4 per emotion) and eight trials consisted of hyperfair offers against an unfair alternative (2 per emotion). The trials including either a hyperfair offer or hyperfair alternative were used to induce more variance in the set of offers and to avoid suspicion from participants being faced with only 5:5 and 8:2 splits on all trials. Therefore, these trials will not be included during analyses (cf. Radke and de Bruijn, 2012).

For analyzing the data, first general rejection behavior was analyzed to investigate the presence of a basic understanding of the task and the concept of fairness. Specifically, rejection rates to fair offers with an unfair alternative and unfair offers with a fair alternative were subjected to a repeated measures ANOVA with fairness (two levels: fair, unfair) and emotion (four levels: angry, happy, neutral, sad) as within-subject factor and group (two levels: schizophrenia patients, healthy controls) as a between-subjects factor.

Second, reactions to unfair offers with the same payoff (8:2) were analyzed against different manipulations of the within-subject factors context and emotion. The factor context refers to the alternative offer that had not been chosen while the factor emotion pertains to the emotional expression of the proposer. The rejection rates were subjected to a repeated measures ANOVA with context (two levels: fair, no alternative) and emotion (four levels: angry, happy, neutral, sad) as within-subject factors and group (two levels: schizophrenia patients, healthy controls) as between subjects-factor.

Separate analyses for possible effects of medication on schizophrenia patients’ rejection behavior alone were also assessed including the chlorpromazine equivalent as a covariate. Within-subject effects of all analyses are reported with Huynh–Feldt corrections in cases were the assumption of Sphericity is violated.

## Results

### Rejection Behavior

**FIGURE 2:**
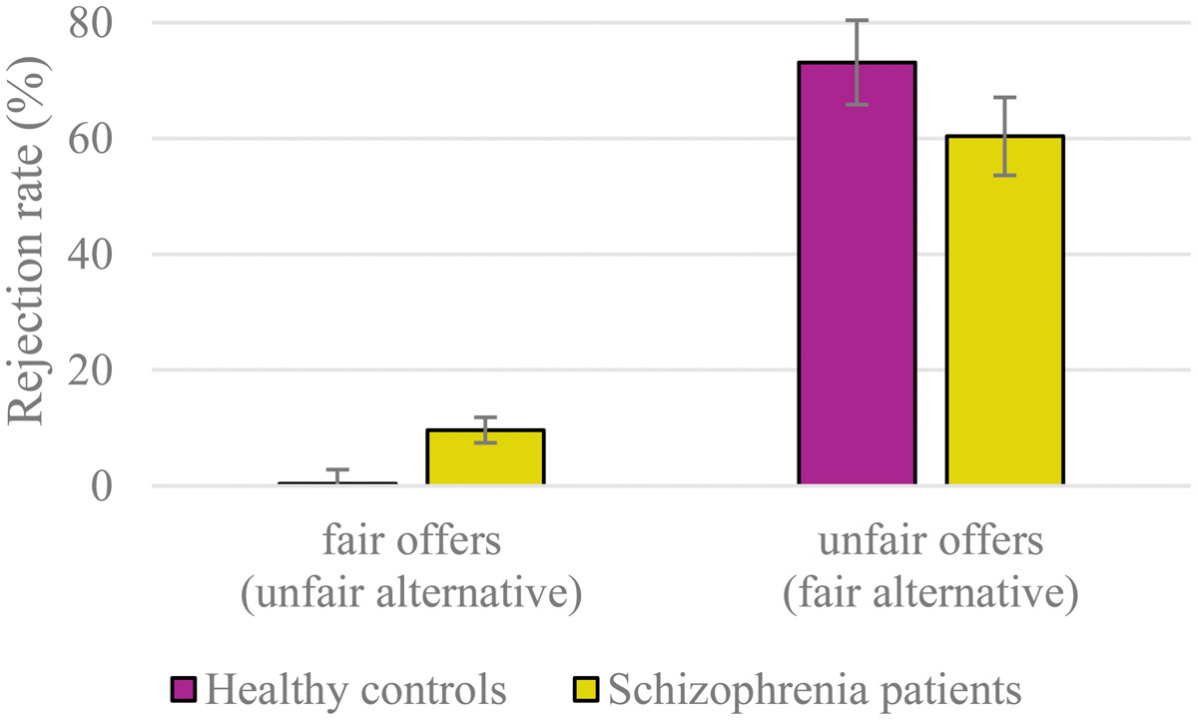
**Rejection rates of fair and unfair offers (collapsed over emotions).** Overall mean percentage and SE are displayed.

The ANOVA repeated measures revealed a significant main effect of fairness [*F*_(1,63)_ = 144.92, *p* < 0.001, η^2^ = 0.70] with higher rejection rates to unfair offers (67%) compared to fair offers (5%). The main effect of emotion was marginally significant [*F*_(1,63)_ = 2.61, *p* = 0.057, η^2^ = 0.04], mainly due to a higher rejection rate of offers from angry proposers (38%) compared to offers from happy proposers (34%; *p* = 0.017). More importantly, the interaction between fairness and group (**Figure 2**) was also significant [*F*_(1,63)_ = 4.60, *p* = 0.036, η^2^ = 0.07]. Follow-up analyses of this interaction showed a significant group difference of the rejection rate to fair offers [*F*_(1,63)_ = 8.02, *p* = 0.006, η^2^ = 0.11], indicating that patients rejected more fair offers (10%) compared to healthy controls (0.4%). Regarding the unfair offers, schizophrenia patients’ acceptance rate (40%) were higher compared to healthy controls’ (27%), however, this difference was only numerical [*F*_(1,63)_ = 1.66, *p* = 0.203, η^2^ = 0.03]. All other main effects and interactions were not significant [All *F*s < 1.61, all *p*s > 0.197, all η^2^ < 0.03].

After separate analyses for the patient group alone including the chlorpromazine equivalent as a covariate, the main effect of fairness remained significant [*F*_(1,33)_ = 28.69, *p* < 0.001, η^2^ = 0.47].

### Reactions to Unfairness

**FIGURE 3:**
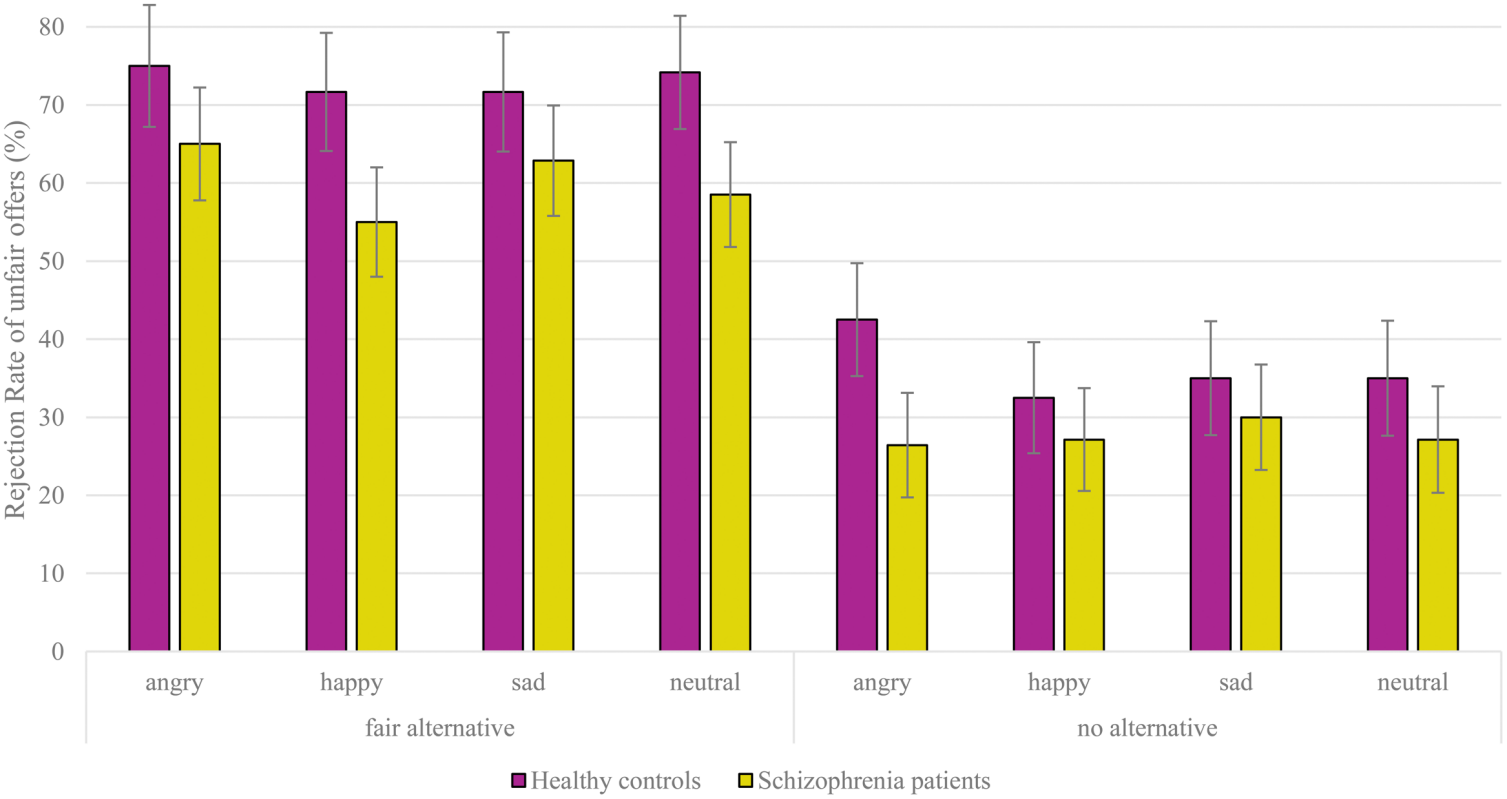
**Rejection rates of unfair offers with regard to the facial emotion of the proposer and alternative offers.** Overall mean percentage and SE of rejections are displayed for schizophrenia patients and healthy individuals.

The results of the ANOVA depicted in **Figure 3** showed a main effect of context [*F*_(1,63)_ = 46.56, *p* < 0.001, η^2^ = 0.43] and emotion [*F*_(3,189)_ = 3.81, *p* = 0.013, η^2^ = 0.06]. The three-way interaction was marginally significant [*F*_(3,189)_ = 2.74, *p* = 0.056, η^2^ = 0.04]. Separate group follow-up analyses revealed a main effect of context in both groups [controls: *F*_(1,29)_ = 22.19, *p* < 0.001, η^2^ = 0.43; patients: *F*_(1,34)_ = 24.24, *p* < 0.001, η^2^ = 0.42] indicated that both healthy controls and schizophrenia patients rejected more unfair offers when the proposer had a fair alternative (controls: 73%; patients: 60%) compared to rejections of unfair offers with no-alternative (controls: 36%; patients: 28%). Importantly however, controls showed a significant effect of emotion [*F*_(3,87)_ = 2.98, *p* = 0.046, η^2^ = 0.09] that was not apparent in the group of patients [*F*_(3,102)_ = 2.13, *p* = 0.109, η^2^ = 0.06]. *Post hoc* pairwise comparisons showed that healthy controls rejected more unfair offers of angry faces (59%) in comparison to unfair offers of happy faces (52%; *p* = 0.016) and neutral faces (55%; *p* = 0.039). Unfair offers of angry faces were also numerically rejected more compared to unfair offers of sad faces (53%; *p* = 0.068). The remaining pairwise comparisons between happy, neutral, and sad faces were not significant (all *p*s > 0.160). Interestingly, when analyzing both groups separately, the patient group also showed a marginal significant two-way interaction between context and emotion [*F*_(3,102)_ = 2.42, *p* = 0.090, η^2^ = 0.07] that was not apparent in the control group [*F* < 1]. Subsequent analyses of this interaction per context revealed that schizophrenia patients showed an effect of emotion only when they were offered an intentional unfair split (fair alternative context) [*F*_(3,102)_ = 3.83, *p* = 0.019, η^2^ = 0.10], but not when they were offered an unintentional unfair split (no-alternative context) [*F*_(3,102)_ < 1]. Follow-up pairwise comparisons showed that during these intentional unfair offers, schizophrenia patients rejected more offers from angry (65%) compared to happy (55%; *p* = 0.021) and neutral proposers (59%; *p* = 0.048), and also rejected more unfair offers from sad (63%) compared to happy proposers (*p* = 0.019). All other main effects or interactions of the primary ANOVA were not significant [All *F*s < 1.68, all *p*s > 0.199, all η^2^ < 0.03].

A closer look at **Figure 3** might imply that while schizophrenia patients are affected by the proposers’ emotions during unfair offers in the context of a fair alternative, healthy controls are rather affected by emotions during unfair offers in the no-alternative context. Therefore, subsequent analyses per context were also executed. The interaction between emotion and group was not significant during the intentional unfair context [*F*_(3,189)_ = 1.46, *p* = 0.228, η^2^ = 0.02], but showed a trend during the unintentional unfair context [*F*_(3,189)_ = 2.36, *p* = 0.073, η^2^ = 0.04]. Follow-up analyses of the latter interaction, suggested that in contrast to schizophrenia patients [*F*_(3,102)_ < 1], only healthy controls [*F*_(3,87)_ = 2.82, *p* = 0.056, η^2^ = 0.09] were affected by the proposers’ emotions during unintentional unfair proposals. More specifically, offers from angry proposers were rejected more (43%) compared to offers from happy (33%; *p* = 0.016), sad (35%; *p* = 0.048), and neutral proposers (35%; *p* = 0.059). This latter result is in line with the previous analyses showing that only healthy controls are affected by emotions, yet particularly when the proposer had no-alternative.

After analyzing the patient group alone including the chlorpromazine equivalents as a covariate, the main effect of context remained significant [*F*_(1,33)_ = 13.37, *p* = 0.001], while there was still no effect of emotion [*F*_(3,99)_ = 1.28, *p* = 0.286].

## Discussion

By using a modified version of the UG in combination with emotional faces we aimed to deepen our understanding of higher-order social decision-making processes involving fairness, intentionality, and emotion considerations in schizophrenia patients. Our primary finding showed that schizophrenia patients and healthy controls were similarly affected by the intentionality behind unfair offers as reflected by the context in which offers were proposed, i.e., more rejections of unfair offers with a fair alternative compared to unfair offers with no-alternative. Second, we found that unlike patients, healthy controls’ decisions to unfair offers were also affected by the emotional state of the proposers (i.e., more rejections of unfair offers from angry proposers compared to unfair offers from happy or neutral proposers). However, subsequent analyses indicated that schizophrenia patients also might be affected by the proposers’ emotions, yet particularly when they were offered an intentional unfair split (i.e., more rejection of intentional unfair offers from angry proposers compared to intentional unfair offers from happy proposers and neutral proposers, and also more rejections of intentional unfair offers from sad versus happy proposers). Third, while both groups rejected unfair offers more often compared to fair offers, schizophrenia patients specifically rejected more fair offers in comparison to healthy controls.

The latter finding is in accordance with our hypothesis stating that schizophrenia patients will show aberrant behavior when considering fair versus unfair offers by either rejecting more fair offers or accepting more unfair offers than healthy controls. This finding is also in line with a previous study of Csukly et al. (2011) who reported that, in comparison to healthy controls, schizophrenia patients rejected more fair offers. Besides this, Agay et al. (2008) also reported that regardless the amount of the offer, schizophrenia patients generally rejected more offers. In contrast, however, Wischniewski and Brüne (2011) did not find increased rejections toward fair offers in schizophrenia patients. Yet, in line with Csukly et al. (2011), they did report the reverse effect where schizophrenia patients accepted more unfair offers, a finding that was not present in the current data. Csukly et al. (2011) suggested that: “rather than being rational maximizers, schizophrenia patients seem to be ‘inconsistent maximizers,’ following a paradox strategy.” A possible explanation for this inconsistent behavior was not directly given by the authors, however, one might assume that because of schizophrenia patients’ heightened state of social anxiety (Green and Phillips, 2004) and tendency to suppress emotions rather than reappraise them (van der Meer et al., 2009), social avoidant behavior is reinforced (de la Asuncion et al., in revision) resulting in more rejections of fair offers. Alternatively, disturbed reward processing or negative symptomatology such as blunted affect and anhedonia might underlie the increased rejection rate of fair offers. Disturbances in reward processing have been demonstrated before in schizophrenia patients and contribute to reduced goal-directed behavior or pleasure-seeking behavior (Strauss et al., 2014) as currently reflected in increased rejection rates of fair offers.

The finding that schizophrenia patients, like healthy controls, reject unfair offers more often in cases where the proposer had the ability to choose for a fair alternative (intentional unfair) compared to cases were the proposer had no alternative (unintentional unfair), are directly in line with our first hypothesis expecting both groups to be sensitive toward the different contexts. Correspondingly, Quisenaerts et al. (2013) reported the same behavior in a group of smoking schizophrenia patients. However, non-smoking schizophrenia patients only showed this pattern after they were administered 1 mg of nicotine. Although we did not register smoking behavior of our participants, we may assume that more than 80% of our patients were smokers (Keltner and Grant, 2006) resembling the smoking group in Quisenaerts et al. (2013) more likely than the non-smoking group. It might therefore be possible that in the current study, patients benefit from the cognitive enhancing properties of nicotine (Newhouse et al., 2011). However, this is merely speculative and must be studied more specifically. Another study using the same paradigm while comparing a group of healthy controls and individuals suffering from depression, also reported more rejections of unfair offers in the context of a fair alternative compared to no alternative in both groups (Radke et al., 2013). Furthermore, previous research in healthy controls found that people responded in a pattern where acceptance rates declines with higher degrees of unfairness (Fehr and Fischbacher, 2003; Sanfey et al., 2003). This pattern was also found in schizophrenia patients (Csukly et al., 2011; Wischniewski and Brüne, 2011) and is in accordance with our finding of both patients and healthy controls rejecting intentional unfair offers more likely compared to unintentional unfair offers. This shows that like healthy individuals, schizophrenia patients adequately recognize unfair intentions and have an intact sense of fairness in general.

Regarding the influence of the proposers’ emotional state while making an offer, we hypothesized that schizophrenia patients would be less sensitive toward the emotion compared to healthy controls. Our results were in line with this proposition and showed that only healthy controls’ behavior was directly influenced by the proposers’ emotions. Specifically, healthy controls rejected unfair distributions more often when they were offered by an angry proposer compared to unfair offers proposed by a happy or a neutral proposer. This is also partially in line with the results of Radke et al. (2013) who reported more rejections of unfair offers from proposers with angry faces compared to happy and sad proposers in both healthy controls and patients with depression. Rejection behavior of schizophrenia patients, however, was not affected by the proposers’ emotions. A similar result was previously reported by Csukly et al. (2014) using a traditional Ultimatum Game. They found that healthy individuals alone accepted slightly unfair offers (40%) and fair offers (50%) more likely when the proposer was happy compared to angry proposers. In the current study, controls were still affected by the proposer’s emotion while they only received an offer of 20% from the total amount. The reason why healthy controls are still likely to accept these highly unfair offers more from happy, neutral, and sad proposers is related to the different contexts, which partially included unintentional unfair offers (no alternative). i.e., since the proposer did not have a real choice when confronted with two equally unfair offers, controls have taken that into account resulting in less rejections of the proposed offer. More importantly, however, is the question why schizophrenia patients were not affected by the proposers’ emotions. One explanation is related to schizophrenia patients’ abnormal emotion-recognition abilities (Edwards et al., 2002). Since these patients have difficulties to distinguish different emotions and are prone to misinterpret ambiguous (de la Asuncion et al., in revision) and neutral emotions (Pinkham et al., 2011), one might argue that these patients do not differentiate well enough between the different emotions and consider them more alike. However, Csukly et al. (2014) controlled for impaired emotion-recognition abilities and did not find an influence of these impairments on the patients’ behavior. Moreover, the currently used task was completely self-paced, providing the participants with ample time to process all the information. A pressure for speed can thus not explain possible integration problems. Therefore, one can also assume that rather than abnormal emotion-recognition abilities in schizophrenia, these patients deviate in the processing of the depicted emotions during this complex task.

This latter assumption is supported by some of the findings during the subsequent analyses we performed. These results showed that healthy controls’ reactions to emotions remained the same during the two unfair contexts with generally more rejections of unfair offers from angry proposers compared to happy, neutral, and sad proposers. Schizophrenia patients, however, showed a marginal significant interaction between emotion and context, implying that these patients’ decisions were only affected by emotions when offered an intentional unfair offer, but not during unintentional unfair offers. First, this indicates that like healthy controls, schizophrenia patients do process the emotions of others during complex social decision-making situations, yet, when focusing on the specific rejection behaviors toward the different emotions, some group differences appear. Specifically, schizophrenia patients reject more intentional unfair offers from sad compared to happy proposers, while both groups reject more (intentional) unfair offers from angry proposers compared to happy and neutral proposers. Possible group differences in attribution style might be responsible for these different reaction patterns toward sad proposers. While healthy controls, for instance, interpret the sad emotion as a sign of compassion toward the participant because of the negative situation (attribution of negative valence to the situation), schizophrenia patients rather attribute this negative emotion to the proposer because of their personalizing bias (attribution of negative valence to the person; Langdon et al., 2006). Alternatively, schizophrenia patients’ impaired Theory of Mind or poor insight of others’ mental states (Brune, 2005), might also be an explanation for their aberrant behavior toward sad proposers. Second, the fact that schizophrenia patients are only affected by the proposers’ emotion during intentional unfair trials, and not during unintentional unfair trials, suggest that these patients process the emotions differently when the offer can be regarded as a genuine unfair choice from the proposer toward the patient. This is in line with previous findings showing that schizophrenia patients who are primed with a negative affective prime, express an exaggerated negative influence on their social judgment (Hooker et al., 2011). Whether the affective prime in this study can be related to the negative emotion or the intentional unfair offer is unclear. However, the fact that schizophrenia patients do not differentiate between emotions when offered an unintentional unfair split, suggest that these patients first process the intentionality of the offer, and depending on how negative the intentions are, further process other contextual factors such as the proposers’ emotions. So when an unfair offer was unintended and the proposer had no real choice, patients seem to feel less affected by it and disregard the proposers’ emotion. Yet, these final interpretations are mainly based on marginal significant results. Therefore, we must remain cautious about their validity, however, in the light of future studies, they might have a significant additional value.

One of the shortcomings of this study is that the participants were mostly males. Therefore, possible gender differences in social decision-making cannot be addressed. However, since the prevalence of schizophrenia is much higher in males than in females (Aleman et al., 2003), the gender differences in the current study represent a rather realistic reflection of general patient populations. Also, all participants in the patient group were on antipsychotic treatment, and we were thus unable to rule out possible effects of antipsychotic medication on patients’ decision-making process. Yet, *post hoc* analyses including patients’ individual chlorpromazine equivalents as a covariate factor, did not change the previously described effects in this group.

Taken together, it is clear that schizophrenia patients adequately differentiate between fair and unfair offers, suggesting that these patients have a basic understanding of moral reasoning. However, when compared directly with healthy individuals, possible disturbances in schizophrenia patients’ reward processing influence their margins of fairness judgments in a way that is disadvantageous for themselves (i.e., higher rejection rates of fair offers). In addition, schizophrenia patients’ ability to differentiate between intentional and unintentional offers is still intact. Both outcomes imply that these social cognitive capacities that play a central role in social decision-making are preserved in schizophrenia. On the other hand, these patients do seem to have problems with processing the emotional information of others during this complex social decision-making task, and even seem to process information differently in different contexts. This shows that schizophrenia patients have problems with correctly combining and integrating different pieces of information during higher order cognitive processes such as social decision-making. In social daily life situations, this might for instance translate in to situations wherein these patients misinterpret, disregard or just incorrectly attribute (personalizing bias), someone’s emotions during complex social situations consisting of multiple contextual elements that also need to be taken into account. This may easily lead to confusion or inappropriate behavior and conflicts. For this reason, the current results emphasize the need for cognitive remediation trainings in patients suffering from schizophrenia in order to enhance not only specific cognitive abilities, but also to improve the integration of different cognitive and affective constructs. Furthermore, the current study also warrants future research aimed at investigating the effects of (social) cognitive training on higher order cognitive processes such as social decision-making, emotion processing, and emotion regulation processes which have proven to be effective in regulating healthy persons’ decisions (van’t Wout et al., 2010) and might help these patients process others’ and own emotions more accurately.

## Author Contributions

Jd and Ed designed the study. Jd and LD wrote the protocol and retrieved all the data. Jd analyzed the data. Jd and Ed wrote the manuscript. LD, MM, and BS contributed to the conception of the work and revised the manuscript critically for important intellectual content. All authors have approved the final manuscript and agree to be accountable for all aspects of the work in ensuring that questions related to the accuracy or integrity of any part of the work are appropriately investigated and resolved.

## Conflict of Interest Statement

The authors declare that the research was conducted in the absence of any commercial or financial relationships that could be construed as a potential conflict of interest.
